# Presence of group II introns in phage genomes

**DOI:** 10.1093/nar/gkaf761

**Published:** 2025-08-13

**Authors:** Liana N Merk, Thomas A Jones, Sean R Eddy

**Affiliations:** Department of Molecular and Cellular Biology, Harvard University, Cambridge, MA 02138, United States; Howard Hughes Medical Institute, Harvard University, Cambridge, MA 02138, United States; Harvard Graduate Program in Biophysics, Harvard University, Cambridge, MA 02138, United States; Department of Molecular and Cellular Biology, Harvard University, Cambridge, MA 02138, United States; Howard Hughes Medical Institute, Harvard University, Cambridge, MA 02138, United States; Department of Molecular and Cellular Biology, Harvard University, Cambridge, MA 02138, United States; Howard Hughes Medical Institute, Harvard University, Cambridge, MA 02138, United States

## Abstract

Although bacteriophage genomes are under strong selective pressure for high coding density, they are still frequently invaded by mobile genetic elements (MGEs). Group II introns are MGEs that reduce host burden by autocatalytically splicing out of an RNA precursor. While widely known in bacterial, archaeal, and eukaryotic organellar genomes, group II introns have been considered absent in phage. Identifying group II introns in genome sequences has previously been challenging because of their lack of primary sequence similarity. Advances in RNA structure-based homology searches using covariance models has provided the ability to identify the conserved secondary structures of group II introns. Here, we discover that group II introns are widely found in phages from diverse phylogenetic backgrounds, from endosymbiont phage to jumbophage.

## Graphical Abstract

**Graphical Abstract:**
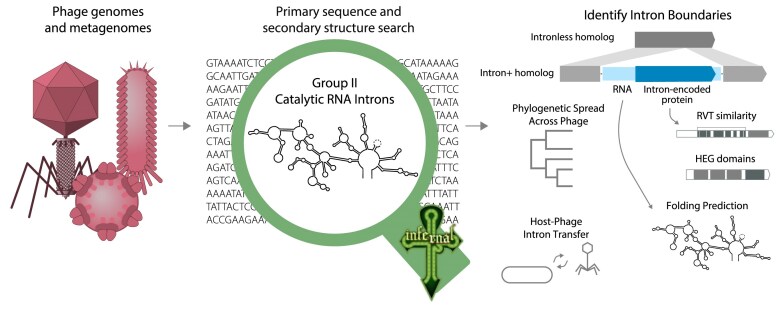

## Introduction

Group II introns are self-splicing ribozymes capable of retromobility [1]. First identified in fungal mitochondrial genomes [2, 3], group II introns have since been identified in all three domains of life: bacteria, archaea, and eukaryotic organelles [4]. They are notably absent in eukaryotic nuclear genomes, though they are likely the ancestral progenitors of spliceosomal introns [1, 5, 6]. Furthermore, despite their wide dispersal in bacterial genomes [7, 8], group II introns have been considered to be absent in phage [9]. Only two examples have been mentioned in passing [10, 11]. Group I introns, in contrast, have long been known in phage genomes and have been studied in depth [12]. One explanation for the apparent disparity between the prevalence of group I introns and the lack of group II introns in phage could be simply that group II introns do exist in phage genomes, but have not been found yet.

**Figure 1.**
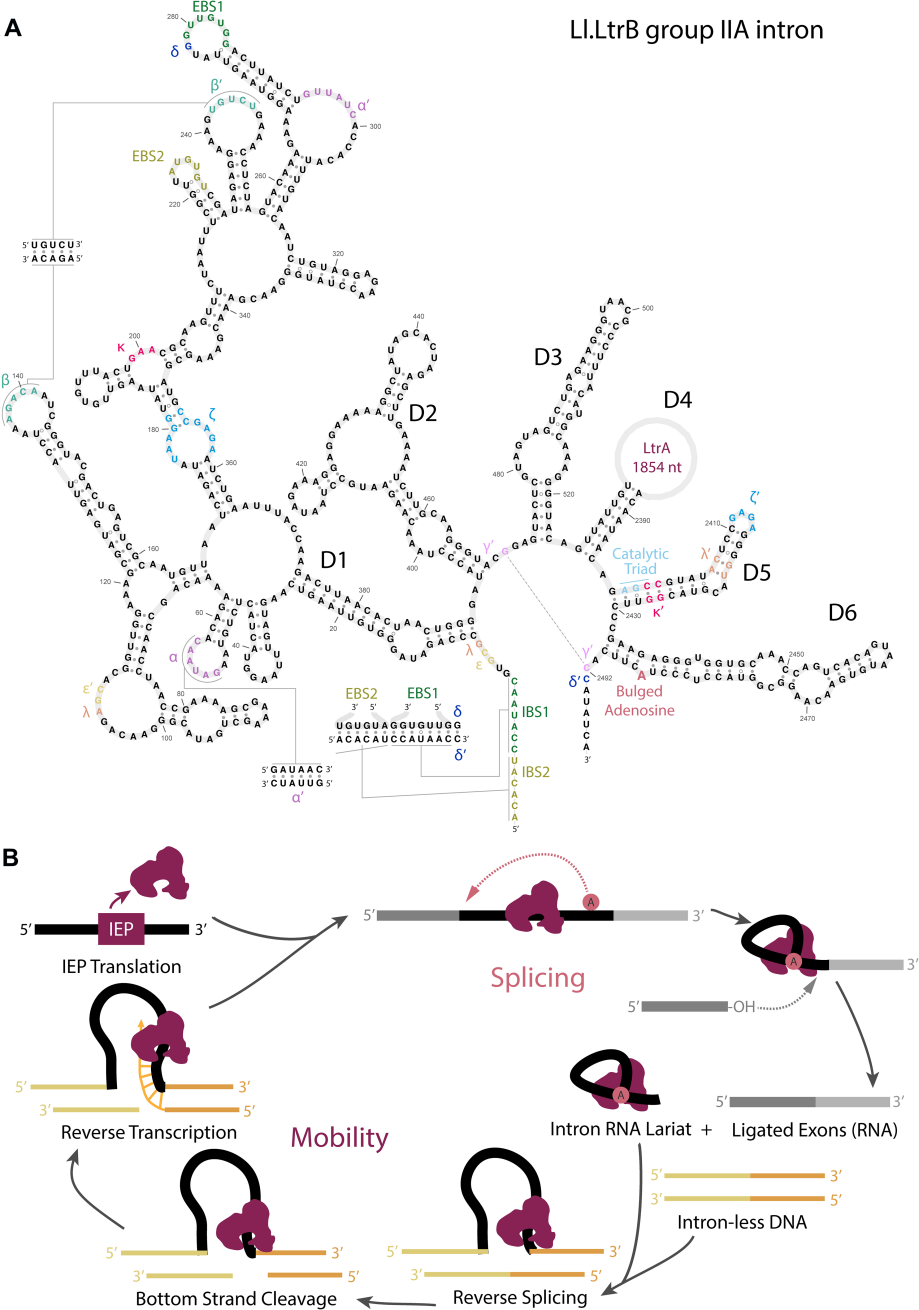
Group II intron consensus secondary structure and mechanisms. (**A**) Secondary structure of *Lactococcus lactis* Ll.LtrB group II intron reproduced from D’Souza *et al.* [13]. The LtrA IEP ORF is located within D4, shown as an open circle. (**B**) Splicing and mobility mechanisms, adapted from [14]. RNA is shown in grey and black, and DNA is shown in yellow and orange. The IEP binds to intron RNA, enabling its folding into splicing-enabled structure. Once the lariat invades intronless DNA, the IEP begins target-primed reverse transcription.

Group II introns are challenging to identify by computational sequence analysis. They have little primary sequence conservation but strong secondary structure conservation. The consensus group II intron secondary structure consists of six domains called D1–D6 (Fig. 1A) [13, 14]. There is often an open reading frame (ORF) for an intron-encoded protein (IEP), typically in the D4 loop. The IEP is usually a multifunctional reverse transcriptase (RVT). ORF-less group II introns are typically around 600 nucleotides in length, and ORF-containing introns are typically around 2–3 kb [15]. The start of the D5 stem contains the catalytic triad (usually AGC or CGC) that coordinates metal ions in the splicing mechanism [4]. The D6 loop contains a bulged adenosine that forms the 2′–5′ branched intermediate. Because D5 and D6 contain the required sequences and structures required for the catalytic splicing mechanism, D5 and D6 are the most conserved part of the group II consensus structure. Previous structure-aware computational efforts to identify new group II introns have focused on these conserved domains [16] or on full-length introns using only primary sequence [15, 17, 18].

Group II introns insert themselves into the DNA of an intronless host gene allele using a “retrohoming” mechanism. A group II intron RVT typically contains an RVT domain, a maturase domain, and an endonuclease domain (Fig. 1B). Homing specificity is determined by base-pairing between intron sequences in D1 and exon sequences at the insertion site, called intron binding site (IBS) and exon binding site (EBS) sequences. With much lower frequency (10^−6^ to 10^−4^), group II introns also transpose to new sites with sufficient IBS–EBS complementarity [19, 20]. Once the intron lariat RNA invades the homing site, the bottom DNA strand is nicked around 10 nucleotides downstream of the insertion site by the endonuclease, forming the primer needed for reverse transcription. Some group II introns only home during replication, using the transient single-stranded DNA (ssDNA) as a primer, in which case the IEP does not include an endonuclease domain [21]. Previous computational search tactics have taken advantage of the fact that IEPs co-evolve with their group II introns [22], and IEPs can be identified by sequence homology. However, relying on IEP homology misses group II introns that are ORF-less or contain non-canonical IEPs, and conversely, there are many RVT homologs that are not associated with group II introns [23, 24].

A general computational method for identifying homologs of a conserved RNA secondary structure and sequence consensus uses probability models called profile stochastic context-free grammars (profile SCFGs, also called covariance models) [25, 26]. A software package called Infernal implements profile SCFG-based search and alignment [27]. The Rfam database of 4000+ known conserved RNA structure elements is built with Infernal [28]. The input to an Infernal search is a multiple sequence alignment of the conserved RNA annotated with its consensus secondary structure. From this sequence and structure information, Infernal builds a consensus statistical model, which can then be used to search genome sequences for homologs, and to structurally align new homologs to the consensus. Profile SCFG algorithms used to be prohibitively computationally expensive, but a set of accelerated algorithms in Infernal now allows for comprehensive searches for RNAs in large genome and metagenome datasets, including RNAs the size of catalytic introns [29]. Here, we use Infernal to search for group II introns in phage genomes.

## Materials and methods

Profile SCFGs for group II introns (RF00029 and CL00102) were downloaded from Rfam 14.10 database [28]. The Millard dataset of 29,015 curated phage genomes was obtained with the INPHARED Perl script [30] on 14 December 2023, and phylogenetic metadata was updated on 11 September 2024. The IMG/VR metagenomic dataset was version 4.1 [31]. Phage genome annotation to aid in determining insertion sites was performed with Bakta 1.9.1 [32] and Pharokka 1.5.1 [33].

Infernal searches used cmsearch from Infernal v1.1.4 (December 2020) with an *E*-value threshold of 0.01. Unannotated IEPs were identified by translating within the intron bounds in three frames, then using hmmscan from HMMER 3.3.2 [34] against Pfam-A 35.0 [35] with an *E*-value cutoff of 10^−3^.

Multiple sequence alignments and phylogenetic tree inference for the RVT domain and terminase large subunit (TerL) were done as follows. Profile hidden Markov models (profile HMMs) for RVT_1 (PF00078) and terminase large subunit (PF03237) were from Pfam 37.0. For RVT, PF00078 was used as an hmmsearch query to identify and align RVT domains from our seven intron-encoded RVT to a set of annotated RVT domain sequences from a dataset from Toro *et al.* [24], randomly subsampling a maximum of 20 RVT domain sequences from each of the five classes (GII, DGR, G2L, Retron-like, and RT/CRISPR–Cas) defined by Toro *et al.* For TerL, PF03237 was used to identify TerL homologs in an iterative two-round hmmsearch of all Millard phage genomes, plus an additional single hmmsearch of a subset of intron-containing IMG/VR genomes that contained a D1–D4 hit and D5/D6 hit within 3 kb of each other. TerL hits in the Millard dataset, which includes phylogenetic classification into 20 families, were randomly sampled to a maximum of 10 TerL sequences per family, for a total of 200. TerL hits in the IMG/VR dataset were single-linkage clustered by pairwise sequence identity with a threshold (17.5%) chosen to result in exactly 30 clusters, then one random sequence was taken from each cluster. The final TerL set consists of 238 sequences: 8 identifiable TerL homologs from our 20 Millard genomes containing candidate introns, 200 representative TerL homologs from phylogenetically annotated intron-negative Millard genomes, and 30 representative TerL homologs from the broader but phylogenetically unannotated IMG/VR genomes. These sets of RVT and TerL protein sequences were then aligned with MAFFT v7.525 [36], and phylogenetic tree inference was done with IQ-TREE2 v2.3.0 with the “model finder plus” flag [37, 38] and 1000 bootstrap replicates. Unrooted trees were visualized with iToL [39]. For all pairwise percent identity values, the denominator is the shorter of the two unaligned sequence lengths.

Secondary structure predictions were done using RNAfold [40] and mfold [41] and visualized using RNAcanvas [42]. Genomic context of introns was plotted using LoVis4u [43].

## Results and discussion

The Rfam database of conserved RNA structure families includes curated multiple sequence alignments and profile SCFGs for 4000+ conserved structural RNAs [28]. Rfam includes eight models of fragments of the group II intron consensus, built from alignments of known eukaryotic organellar and bacterial group II intron sequences. The most conserved region of the intron is the 3′ D5/D6 region, Rfam model RF00029. The 5′ end of the intron, domains D1–D4, varies widely across group II intron classes and is represented in Rfam by a set of seven models grouped into an Rfam “clan,” CL00102. For each complete group II intron, we expect to find one or two hits: a D1–D4 hit to one of the seven 5′ end models, and a D5/D6 hit a few hundred nucleotides or around 1 kb downstream, for an ORF-less and ORF-containing intron, respectively.

Available phage genome sequences have grown rapidly in recent years, collected in different datasets. We started with a well-curated phage genome dataset provided by the Millard lab, comprising 29,015 phage genomes totaling 1.77 Gb. Using the Infernal search program cmsearch, we found 27 hits to the D1–D4 models and 23 hits to the D5/D6 model in the Millard phage genomes with *E*-value < 0.01.

Looking at these hits, we removed four genomes (MK448731, MK448731, MK448781, MK448888) where putative intron regions were identical to other representative genomes; one genome (NC_030940) where a group II intron occurs just before the prophage integration site and appears to be misannotated as being within the prophage; and three scaffolds that appear to be integrated conjugative elements (MT836071, MT836602, MT836027). We kept both of two other group II introns that are identical in sequence (in NC_031039 and NC_043027), because both phages (AR9 and PBS1) have been well studied, and the host RNA polymerase gene and a downstream group I intron in it are not identical.

**Figure 2.**
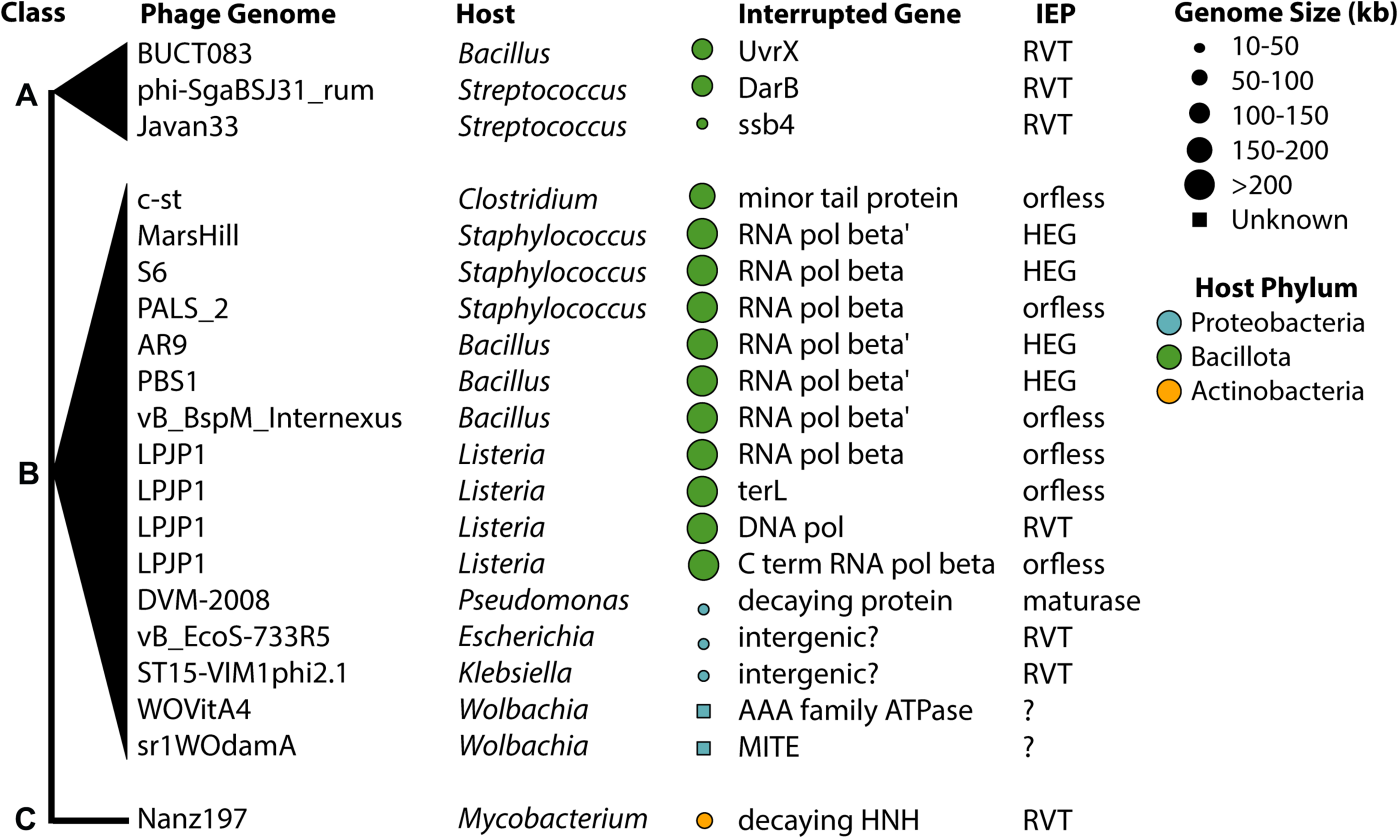
Phage group II introns. Genome size is indicated by size of circle to right of genome name, and host bacterial phylum by color. Two genomes were incomplete contigs (WOVitA4 and sr1WOdamA) and thus their genome size bubble is denoted as a box (for unknown). Introns are sorted by class: IIA-, IIB-, or IIC-type introns.

After these removals, we had a set of 20 putative group II introns in phage genomes in the Millard database (Fig. 2). Of these, 12 have both D1–D4 and D5/D6 hits, 4 have D1–D4 and no D5/D6, and 4 have only a D5/D6 hit. One intron hit, OQ555808, contained two D1-D4 hits: to model group-II-D1D4-3, followed by group-II-D1D4-1. Upstream and downstream hits, when both present, were always within 2 kb of each other, as expected for typical group II intron length.

We then used cmsearch with the same eight Rfam models to search the much larger IMG/VR metagenomic viral database of 15.7 million contigs and found putative intron hits in 9,229 genomes: 6,205 hits to the D1–D4 models and 6,703 to the D5/D6 model with *E*-value < 0.01 (Supplementary Table S2). The proportion of intron-containing genomes was approximately the same between Millard (20 of 29,015 = 0.07%) and IMG/VR (9,229 of 15,722,824 = 0.06%). The IMG/VR phage hits included an unusual one worth noting: contig IMGVR_UViG_3300011577_000009 is annotated as a high-confidence *Inovirus*, a ssDNA phage. The group II intron mobility mechanism targets double-stranded DNA (dsDNA), and no ssDNA genomes with group II introns have been described previously. We used hmmscan and Pfam to confirm that this phage genome contig contains homologs distinctive of *Inoviridae*, a Zot domain and a replication G2P protein. The intron itself appears to be complete, intact, and contains a clear RVT. We imagine that an ssDNA virus could be infected by a group II intron during replication, when it exists in a dsDNA intermediate.

We searched with additional profile SCFGs we built from the newly identified phage introns, to account for the possibility that the existing Rfam models might imperfectly represent their sequence diversity. We made a new profile SCFG of the alignment of 6703 D5/D6 hits in IMG/VR hits (using the cmsearch -A flag), then re-searched IMG/VR using this alignment. This identified hits in another 680 IMG/VR contigs besides the previous 9,229. We made two full-length group II intron models, one of the 3 type IIA and the other of the 16 type IIB introns (Fig. 2), starting from our inferred structures for ON107264 and NC_007581, respectively. These models identified 711 and 97 new hits in IMG/VR. These results indicated that more refined and/or phage-specific models could reveal some additional group II introns, but the gains from the effort seemed incremental. None of the three phage-specific models identified additional candidate introns in the genomes in the Millard dataset.

We used three additional lines of evidence, in addition to significant Infernal search results, to improve our confidence in the set of 20 putative group II introns. First, we sought to identify closely related intronless homologs in other phage genomes, to confirm that candidate introns are discrete intervening sequences in comparisons of intron-plus versus intron-minus loci. We stitched together flanking exons to infer the host gene protein sequence, and then used this to identify intronless homologs in other phages. We also used the alignment to the closest intronless homolog to help define the 5′ and 3′ intron boundaries (Supplementary Table S1). The 3′ splice site is generally closely determined by the hit to the conserved D5/D6 model, and the 5′ splice site occurs at a conserved GWYRG site [4, 44] in the upstream vicinity of the hits to Rfam D1–D4 models. Fourteen of the 20 candidate introns are in intervening sequences in host gene coding regions where we could identify and align to an intronless homolog. Of the remaining six, two appear to be intergenic (ON470608, MK448228), one appears in a heavily decayed pseudogene (EU982300), two are incomplete intron sequences with start or end outside of the contig boundaries (HQ906664, KY695241), and one is downstream of a rho-independent terminator in a typical position for bacterial group IIC introns (OQ555808; Supplementary Fig. S1) [45]. Although we did not confirm splicing activity experimentally, introns interrupting coding regions of conserved phage proteins suggests that these introns must be active.

**Figure 3.**
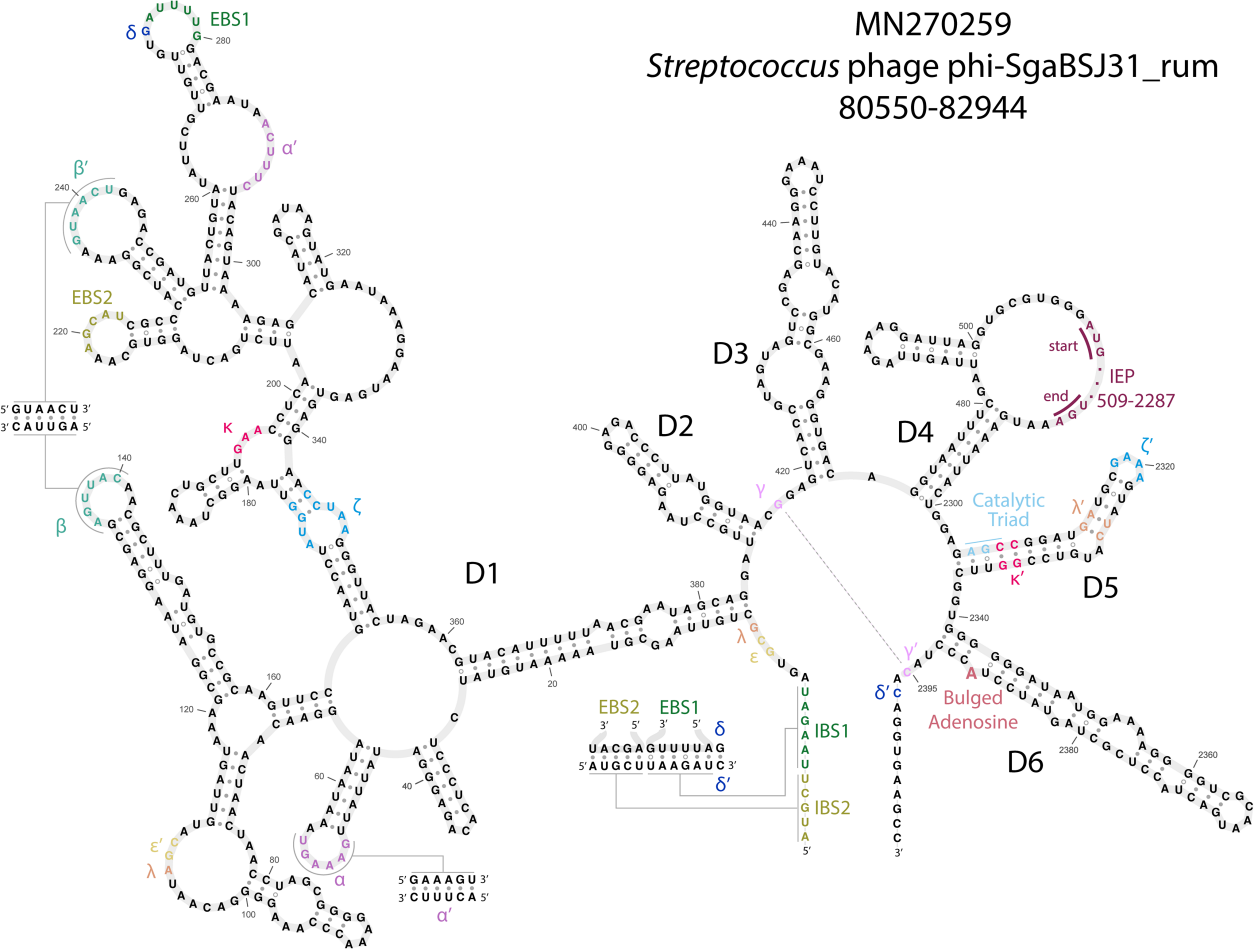
Secondary structure example. Manually curated predicted secondary structure of the group IIA intron in a DarB-like antirestriction/SNF2 helicase gene in *Streptococcus* phage phi-SgaBSJ31_rum.

Second, we identified a set of consensus pseudoknotted base-pairing interactions critical for group II intron function using secondary structure prediction tools and manual curation. Because profile SCFG algorithms do not model pseudoknots, these additional base-pairing interactions provide independent evidence in support of a group II intron call. Specifically, we looked for pseudoknot base-pairing interactions EBS1:IBS1, EBS2:IBS2, α:α′, β:β′, δ:δ′, ε:ε′, and γ:γ′, and also for tertiary non-canonical pairing interactions λ:λ′, κ:κ′, and ζ:ζ′. Figure 3 shows an example structure of one of the phage introns, with these interactions annotated. We used the region around λ − ε′ to classify the group II introns by subtype, where A has an 11-nt loop with consensus AGC, B is a 4-nt bulge with consensus AARC, and C contains a 7–12-nt loop with consensus AGG [2, 44]. To confirm the class affiliation of each intron, we use primary sequence similarity to previously classified bacterial introns using the Zimmerly and Candales database [46]. Examples of all three classes were identified (Fig. 2).

**Figure 4.**
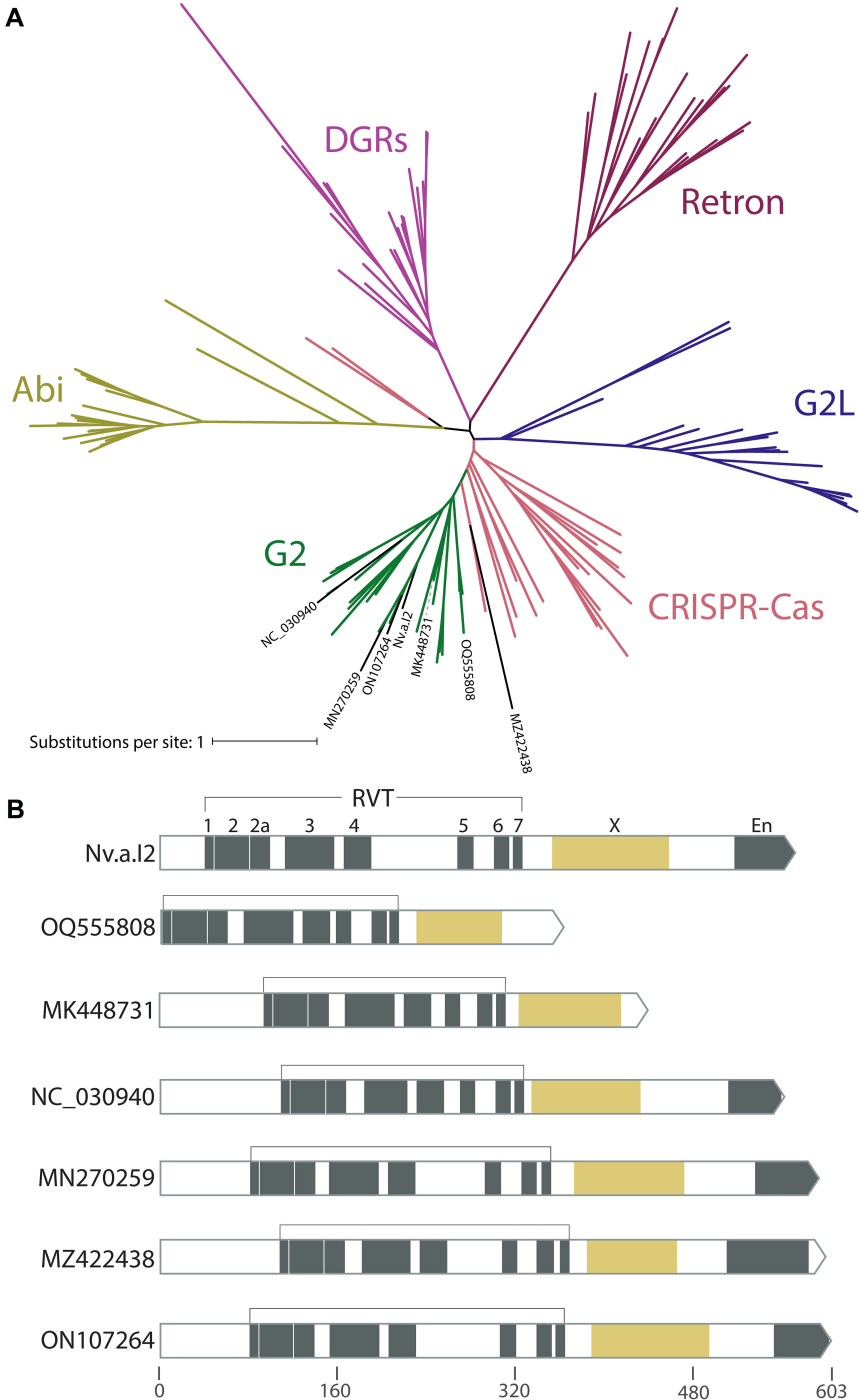
RVT phylogeny. (**A**) Unrooted tree of RVTs found in various genetic elements in bacteria and phage. RVT domains of known group II intron IEPs are shown in green, and RVT domains of phage group II IEPs identified in this paper labeled in black. Abi, abortive infection defense systems; DGRs, diversity generating elements; G2L, “group II-like”. (**B**) Domain structure of phage group II IEPs includes the maturase “X” domain characteristic of group II IEPs, and the frequent presence of an endonuclease domain. For comparison, domain structure of a known group II IEP is shown at top (Nv.a.I2, a bacterial group IIA intron in *Novosphingobium aromaticivorans* [46]).

Lastly, we analyzed ORFs encoded by the 20 group II introns. We translated the regions within the intron to identify a total of seven RVTs and four homing endonucleases (HEGs) (Fig. 2 and Supplementary Table S1). Many types of RVTs occur in bacterial and phage genomes, including retrons, CRISPR–Cas systems, diversity generating retroelements, and phage defense systems [24, 47–49]. The seven intron-encoded RVTs were generally incorrectly classified in GenBank files, often as “retron-type” RVTs, a broader issue known in the field [50, 51]. Phylogenetic tree inference placed all seven RVTs within the clade of known group II intron-encoded RVT to the exclusion of other known RVT clades including retrons (Fig. 4A). All seven RVTs also have the “X” maturase domain characteristic of a group II intron-encoded RVT (Fig. 4B).

Six introns are ORF-less, but this does not necessarily imply that they are immobile. An ORF-less phage group II intron may retain RVT-mediated mobility if another group II intron in the same cell encodes an RVT *trans* [52]. For example, one phage genome (LPJP1, a *Listeria* jumbophage) contains four group II introns, one of which encodes an RVT IEP, and three of which are ORF-less (Fig. 2). Because these LPJP1 introns are structurally related, albeit with low (40%–50%) nucleotide identity, we speculate the RVT encoded by one intron could act in *trans* to mobilize the three ORF-less introns.

**Figure 5.**
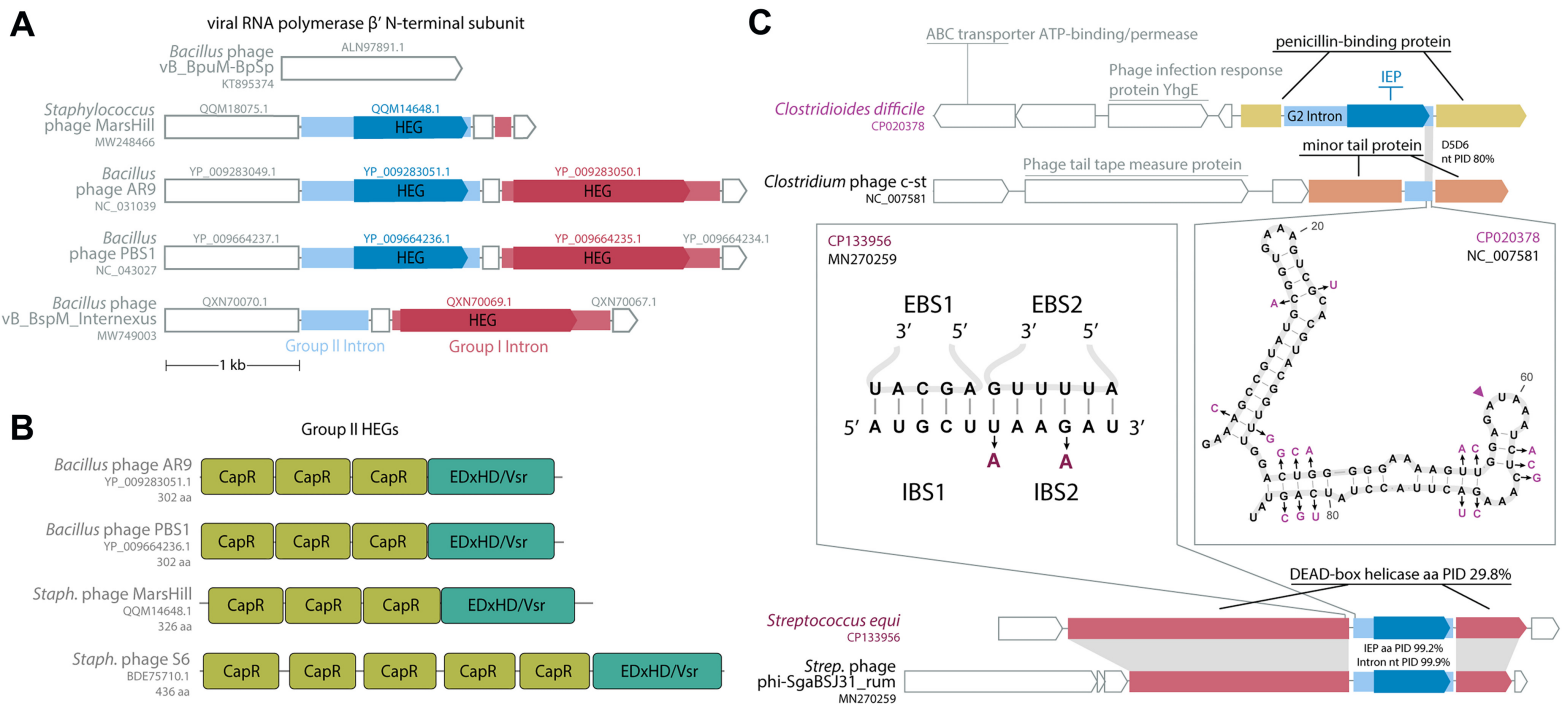
Homing endonuclease genes found in four introns; close bacterial relatives found for two. (**A**) Phage genes for the virion RNA polymerase β′ N-terminal subunit are invaded by both group I and group II introns with homologous HEGs. Group I introns are shown in red, with their encoded HEGs shown in darker red. Group II introns are shown in light blue, with their encoded HEG in darker blue. (**B**) Four group II introns [including three shown in panel (A)] encode a large new outgroup of HEGs which consist of several domains distant related to Pfam CapR domains (light green) followed by a putative nuclease domain distantly homologous to EDxHD/Vsr homing enconucleases (teal). (**C**) Two phage group II introns have closely related bacterial homologs. Phage loci are labeled in black; host loci are labeled in purple. One case (top diagram and right panel) is a possible retroposition event between unrelated host genes. The other case (bottom diagram and left panel) involves homologous host genes in the phage and bacterial genomes. The structure inset (right panel) shows the D5/D6 region of the phage intron, and substitutions in the bacterial homolog are shown in purple.

We looked closely at the four intron ORFs that appear to encode homing endonucleases instead of RVT. HEGs are more typical of mobile group I introns, and indeed we find additional homologs of these four HEGs elsewhere in the same phage genomes and mostly in group I introns, including a case of HEG-containing group I and group II introns in the same host gene (Fig. 5A). HEG-based mobility works by the HEG making a specific dsDNA cleavage at the intron insertion site in an intronless allele, which is then a substrate for double-strand break repair recombination using the intact intron-plus allele as the repair template. Unlike the group II intron retrohoming mechanism, HEG-mediated mobility is a DNA-level event independent of RNA catalysis, so HEGs are found in many types of mobile sequences, including group I introns, bulge–helix–bulge introns, and inteins, and free-standing mobile intergenic HEGs are also common [53]. A few cases of group II introns with HEGs have been observed before, for two of the most abundant HEG families, the LAGLIDADG and GIY-YIG HEGs [5, 54]. These four phage group II HEGs are not closely related to known HEG families. HMMER profile analysis and AlphaFold3 structure prediction [55] identify a conserved domain structure with three to five ∼60aa domains that are distantly related to Pfam models CapR, DUF4379, and DUF723, followed by a C-terminal ∼100aa domain distantly related to the known EDxHD/Vsr HEG endonuclease domain [56] (Fig. 5B). The CapR-like domain is likely to be a DNA-binding domain that confers additional sequence specificity onto the HEG endonuclease domain, as seen with several known families of auxiliary “NUMODs” (nuclease-associated modular DNA-binding domains) [57]. Profile HMMs for these CapR-like and EDxHD/Vsr-like domains find thousands of hits in UniProt, many of which are unannotated hypothetical proteins, so this appears to be a large unrecognized outgroup of the EDxHD/Vsr HEGs.

One way for a phage genome to acquire group II introns is by retroposition from the host bacterial genome. This would be more likely to occur for lysogenic phage with an integrated prophage stage in their life cycle, as opposed to lytic phage. We searched for bacterial group II introns similar to our phage introns and identified two examples (Fig. 5C). In both cases, the intron-containing phage is annotated as a lysogenic prophage. For the group II intron in prophage c-st, we found a diverged host intron (∼80% identical in D5/D6) in a nonhomologous host gene. For the intron in prophage phi-SgaBSJ31_rum, we found a near-identical intron in a helicase gene remotely homologous to the phage host gene, a DEAD-box helicase annotated as a DarB antirestriction system. The immediate exonic flanking regions of the phage and bacterial introns are similar, with two substitutions in IBS2, one of which conserves pairing, and the other of which is the dispensable first position of IBS2 (since EBS2:IBS2 can be as short as 4 bp). Although retroposition is rare (orders of magnitude less efficient than retrohoming) and we cannot rule out other methods of acquisition, we speculate these two examples may represent possible retroposition events from their bacterial hosts.

**Figure 6.**
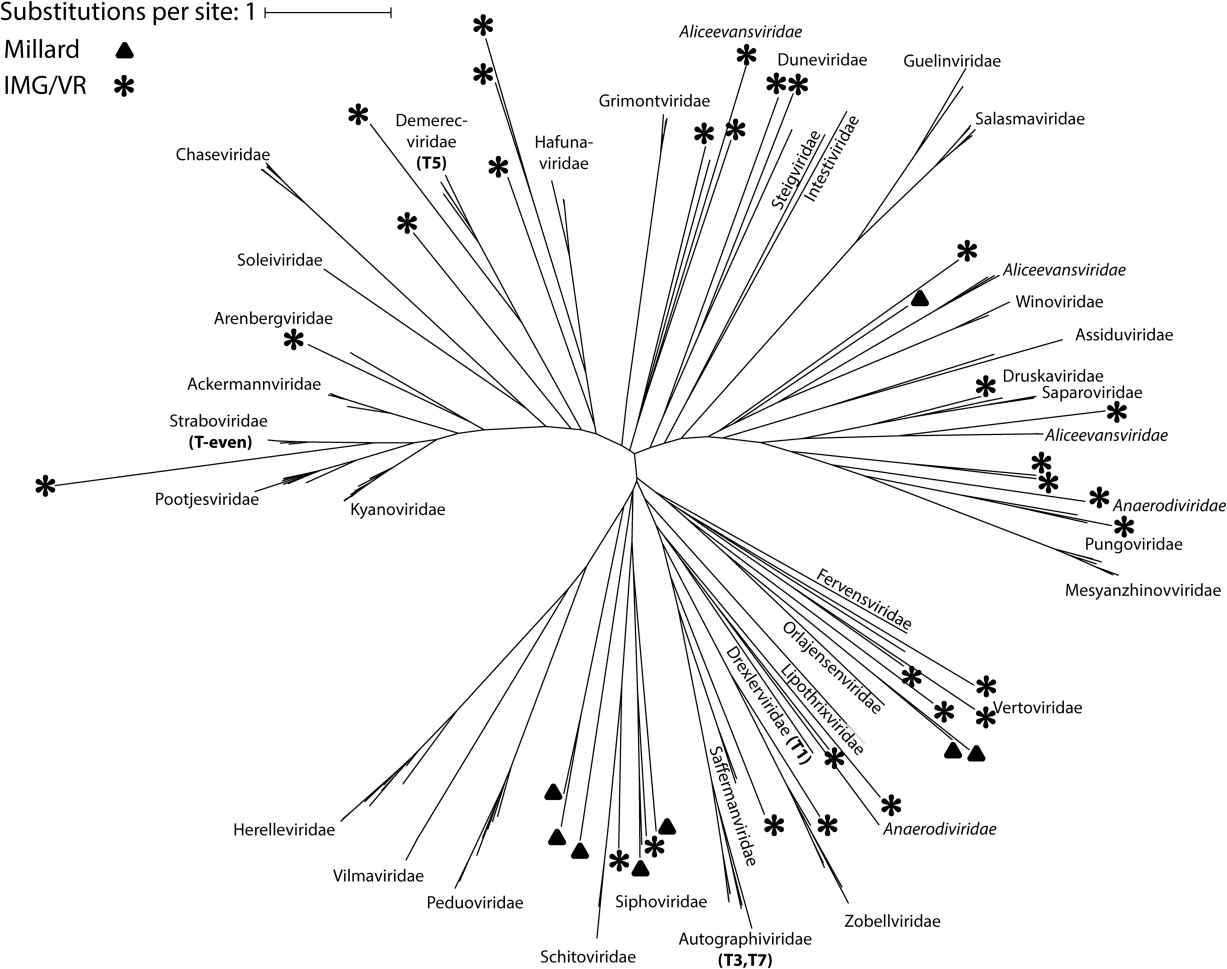
Phylogenetic distribution of phage group II introns. Unrooted tree using an alignment of terminase large subunit (TerL) protein sequences, with phage families labeled. Italics indicating a polyphyletic family in the tree. Taxa containing group II introns are denoted with a black triangle (Millard dataset) or star (IMG/VR).

We sought to determine whether group II introns are widespread across different types of phages, or whether they are confined to a particular phylogenetic clade. While there is no universal phylogenetic marker for phage, all 20 of our examples from the Millard dataset (and most of the IMG/VR hits) are within *Caudoviricetes*, for which the terminase large subunit TerL is often used for taxonomic classification [58, 59]. A phylogenetic tree inferred from an alignment of TerL protein sequences from both intron-plus and intron-minus representatives across all families represented in the Millard database shows that group II introns are dispersed across *Caudoviricetes* phage phylogeny (Fig. 6).

Group I introns have been known to occur as mobile genetic elements in phage for many years [9]. The sporadic occurrence of group I introns, with apparent preferences for particular genes involved in nucleotide synthesis and DNA metabolism, has remained mysterious [9]. The presence of group II introns in a wide variety of different phages means that both types of self-splicing introns have invaded phage genomes, sometimes using the same HEG-based homing mechanism. Comparison of group I and group II intron distribution may help shed more light on what evolutionary dynamics promote and restrict their occurrence.

Besides phage, another place that group II introns are thought to be absent is eukaryotic nuclear genomes. Using the latest accelerated versions of Infernal, systematic searches of all eukaryotic nuclear genomes with group II intron profile SCFGs are feasible.

## Supplementary Material

gkaf761_Supplemental_Files

## Data Availability

Code and data are available on Zenodo: doi.org/10.5281/zenodo.15490104.

## References

[1] Lambowitz AM, Belfort M Mobile bacterial group II introns at the crux of eukaryotic evolution. Microbiol Spect. 2015; 3:MDNA3-0050-–2014. 10.1128/microbiolspec.MDNA3-0050-2014 PMC4394904 26104554

[2] Michel F, Jacquier A, Dujon B Comparison of fungal mitochondrial introns reveals extensive homologies in RNA secondary structure. Biochimie. 1982; 64:867–81. 6817818 10.1016/s0300-9084(82)80349-0

[3] Michel F, Dujon B Conservation of RNA secondary structures in two intron families including mitochondrial-, chloroplast- and nuclear-encoded members. EMBO J. 1983; 2:33–8. 11894905 10.1002/j.1460-2075.1983.tb01376.x PMC555082

[4] Zimmerly S, Semper C Evolution of group II introns. Mobile DNA. 2015; 6:7. 25960782 10.1186/s13100-015-0037-5 PMC4424553

[5] Lambowitz AM, Zimmerly S Group II Introns: mobile ribozymes that invade DNA. Cold Spring Harb Perspect Biol. 2011; 3:a003616. 20463000 10.1101/cshperspect.a003616 PMC3140690

[6] Smathers CM, Robart AR The mechanism of splicing as told by group II introns: ancestors of the spliceosome. Biochim Biophys Acta Gene Regul Mech. 2019; 1862:194390. 31202783 10.1016/j.bbagrm.2019.06.001

[7] Miura MC, Nagata S, Tamaki S et al. Distinct expansion of group II introns during evolution of prokaryotes and possible factors involved in its regulation. Front Microbiol. 2022; 13:849080. 35295308 10.3389/fmicb.2022.849080 PMC8919778

[8] Toro N, Jiménez-Zurdo JI, García-Rodríguez FM Bacterial group II introns: not just splicing. FEMS Microbi Rev. 2007; 31:342–58. 10.1111/j.1574-6976.2007.00068.x 17374133

[9] Edgell DR, Belfort M, Shub DA Barriers to intron promiscuity in bacteria. J Bacteriol. 2000; 182:5281–9. 10986228 10.1128/jb.182.19.5281-5289.2000 PMC110968

[10] Lavysh D, Sokolova M, Minakhin L et al. The genome of AR9, a giant transducing *Bacillus* phage encoding two multisubunit RNA polymerases. Virology. 2016; 495:185–96. 27236306 10.1016/j.virol.2016.04.030 PMC13189113

[11] Korn AM, Hillhouse AE, Sun L et al. Comparative genomics of three novel jumbo bacteriophages infecting *Staphylococcus aureus*. J Virol. 2021; 95:e0239120. 34287047 10.1128/JVI.02391-20 PMC8428398

[12] Hausner G, Hafez M, Edgell DR Bacterial group I introns: mobile RNA catalysts. Mobile DNA. 2014; 5:8. 24612670 10.1186/1759-8753-5-8 PMC3984707

[13] D’Souza LM, Zhong J Mutations in the *Lactococcus lactis* Ll.LtrB group II intron that retain mobility *in vivo*. BMC Mol Biol. 2002; 3:17. 12495443 10.1186/1471-2199-3-17 PMC151599

[14] Belfort M, Lambowitz AM Group II intron RNPs and reverse transcriptases: from retroelements to research tools. Cold Spring Harb Perspect Biol. 2019; 11:a032375. 30936187 10.1101/cshperspect.a032375 PMC6442199

[15] Dai L, Zimmerly S Compilation and analysis of group II intron insertions in bacterial genomes: evidence for retroelement behavior. Nucleic Acids Res. 2002; 30:1091–102. 11861899 10.1093/nar/30.5.1091 PMC101233

[16] Lang BF, Laforest MJ, Burger G Mitochondrial introns: a critical view. Trends Genet. 2007; 23:119–25. 17280737 10.1016/j.tig.2007.01.006

[17] Zimmerly S, Hausner G, chu Wu X Phylogenetic relationships among group II intron ORFs. Nucleic Acids Res. 2001; 29:1238–50. 11222775 10.1093/nar/29.5.1238 PMC29734

[18] Dai L, Toor N, Olson R et al. Database for mobile group II introns. Nucleic Acids Res. 2003; 31:424–6. 12520040 10.1093/nar/gkg049 PMC165496

[19] Coros CJ, Landthaler M, Piazza CL et al. Retrotransposition strategies of the *Lactococcus lactis* Ll.LtrB group II intron are dictated by host identity and cellular environment. Mol Microbiol. 2005; 56:509–24. 15813740 10.1111/j.1365-2958.2005.04554.x

[20] Ichiyanagi K, Beauregard A, Lawrence S et al. Retrotransposition of the Ll.LtrB group II intron proceeds predominantly via reverse splicing into DNA targets. Mol Microbiol. 2002; 46:1259–72. 12453213 10.1046/j.1365-2958.2002.03226.x

[21] García-Rodríguez FM, Neira JL, Marcia M et al. A group II intron-encoded protein interacts with the cellular replicative machinery through the -sliding clamp. Nucleic Acids Res. 2019; 47:7605–17. 31127285 10.1093/nar/gkz468 PMC6698660

[22] Toor N, Hausner G, Zimmerly S Coevolution of group II intron RNA structures with their intron-encoded reverse transcriptases. RNA. 2001; 7:1142–52. 11497432 10.1017/s1355838201010251 PMC1370161

[23] Mestre MR, Gao LA, Shah SA et al. UG/Abi: a highly diverse family of prokaryotic reverse transcriptases associated with defense functions. Nucleic Acids Res. 2022; 50:6084. 35648479 10.1093/nar/gkac467 PMC9226505

[24] Toro N, Martínez-Abarca F, Mestre MR et al. Multiple origins of reverse transcriptases linked to CRISPR–Cas systems. RNA Biol. 2019; 16:1486–93. 31276437 10.1080/15476286.2019.1639310 PMC6779382

[25] Eddy SR, Durbin R RNA sequence analysis using covariance models. Nucleic Acids Res. 1994; 22:2079–88. 8029015 10.1093/nar/22.11.2079 PMC308124

[26] Sakakibara Y, Brown M, Hughey R et al. Stochastic context-free grammars for tRNA modeling. Nucleic Acids Res. 1994; 22:5112–20. 7800507 10.1093/nar/22.23.5112 PMC523785

[27] Nawrocki EP, Eddy SR Infernal 1.1: 100-fold faster RNA homology searches. Bioinformatics. 2013; 29:2933–5. 24008419 10.1093/bioinformatics/btt509 PMC3810854

[28] Kalvari I, Nawrocki EP, Ontiveros-Palacios N et al. Rfam 14: expanded coverage of metagenomic, viral and microRNA families. Nucleic Acids Res. 2021; 49:D192–200. 33211869 10.1093/nar/gkaa1047 PMC7779021

[29] Nawrocki EP, Jones TA, Eddy SR Group I introns are widespread in archaea. Nucleic Acids Res. 2018; 46:7970–6. 29788499 10.1093/nar/gky414 PMC6125680

[30] Cook R, Brown N, Redgwell T et al. INfrastructure for a PHAge reference database: identification of large-scale biases in the current collection of cultured phage genomes. PHAGE. 2021; 2:214–23. 36159887 10.1089/phage.2021.0007 PMC9041510

[31] Camargo AP, Nayfach S, Chen IMA et al. IMG/VR v4: an expanded database of uncultivated virus genomes within a framework of extensive functional, taxonomic, and ecological metadata. Nucleic Acids Res. 2023; 51:D733–43. 36399502 10.1093/nar/gkac1037 PMC9825611

[32] Schwengers O, Jelonek L, Dieckmann MA et al. Bakta: rapid and standardized annotation of bacterial genomes via alignment-free sequence identification. Microb Genom. 2021; 7:000685. 34739369 10.1099/mgen.0.000685 PMC8743544

[33] Bouras G, Nepal R, Houtak G et al. Pharokka: a fast scalable bacteriophage annotation tool. Bioinformatics. 2023; 39:btac776. 36453861 10.1093/bioinformatics/btac776 PMC9805569

[34] Eddy SR Accelerated profile HMM searches. PLoS Comput Biol. 2011; 7:e1002195. 22039361 10.1371/journal.pcbi.1002195 PMC3197634

[35] Mistry J, Chuguransky S, Williams L et al. Pfam: The protein families database in 2021. Nucleic Acids Res. 2021; 49:D412–9. 33125078 10.1093/nar/gkaa913 PMC7779014

[36] Katoh K, Standley DM MAFFT multiple sequence alignment software version 7: improvements in performance and usability. Mol Biol Evol. 2013; 30:772–80. 23329690 10.1093/molbev/mst010 PMC3603318

[37] Minh BQ, Schmidt HA, Chernomor O et al. IQ-TREE 2: new models and efficient methods for phylogenetic inference in the genomic era. Mol Biol Evol. 2020; 37:1530–4. 32011700 10.1093/molbev/msaa015 PMC7182206

[38] Kalyaanamoorthy S, Minh BQ, Wong TKF et al. ModelFinder: fast model selection for accurate phylogenetic estimates. Nat Methods. 2017; 14:587–9. 28481363 10.1038/nmeth.4285 PMC5453245

[39] Letunic I, Bork P Interactive tree of life (iTOL) v6: recent updates to the phylogenetic tree display and annotation tool. Nucleic Acids Res. 2024; 52:W78–82. 38613393 10.1093/nar/gkae268 PMC11223838

[40] Gruber AR, Lorenz R, Bernhart SH et al. The vienna RNA websuite. Nucleic Acids Res. 2008; 36:W70–4. 18424795 10.1093/nar/gkn188 PMC2447809

[41] Zuker M Mfold web server for nucleic acid folding and hybridization prediction. Nucleic Acids Res. 2003; 31:3406–15. 12824337 10.1093/nar/gkg595 PMC169194

[42] Johnson PZ, Simon AE RNAcanvas: interactive drawing and exploration of nucleic acid structures. Nucleic Acids Res. 2023; 51:W501–8. 37094080 10.1093/nar/gkad302 PMC10320051

[43] Egorov AA, Atkinson GC LoVis4u: a locus visualization tool for comparative genomics and coverage profiles. NAR Genom Bioinform. 2025; 7:lqaf009. 40007724 10.1093/nargab/lqaf009 PMC11850299

[44] Michel F, Kazuhiko U, Haruo O Comparative and functional anatomy of group II catalytic introns—a review. Gene. 1989; 82:5–30. 2684776 10.1016/0378-1119(89)90026-7

[45] Robart AR, Seo W, Zimmerly S Insertion of group II intron retroelements after intrinsic transcriptional terminators. PNAS. 2007; 104:6620–5. 17420455 10.1073/pnas.0700561104 PMC1871835

[46] Candales MA, Duong A, Hood KS et al. Database for bacterial group II introns. Nucleic Acids Res. 2012; 40:D187–90. 22080509 10.1093/nar/gkr1043 PMC3245105

[47] Wilkinson ME, Li D, Gao A et al. Phage-triggered reverse transcription assembles a toxic repetitive gene from a noncoding RNA. Science. 2024; 386:eadq3977. 39208082 10.1126/science.adq3977 PMC12039810

[48] Tang S, Conte V, Zhang DJ et al. *De novo* gene synthesis by an antiviral reverse transcriptase. Science. 2024; 386:eadq0876. 39116258 10.1126/science.adq0876 PMC11758365

[49] González-Delgado A, Mestre MR, Martínez-Abarca F et al. Prokaryotic reverse transcriptases: from retroelements to specialized defense systems. FEMS Microbiol Rev. 2021; 45:fuab025. 33983378 10.1093/femsre/fuab025 PMC8632793

[50] Sharifi F, Ye Y Identification and classification of reverse transcriptases in bacterial genomes and metagenomes. Nucleic Acids Res. 2022; 50:e29. 34904653 10.1093/nar/gkab1207 PMC8934634

[51] Toro N, Nisa-Martínez R Comprehensive phylogenetic analysis of bacterial reverse transcriptases. PLoS One. 2014; 9:e114083. 25423096 10.1371/journal.pone.0114083 PMC4244168

[52] Meng Q, Wang Y, Liu XQ An intron-encoded protein assists RNA splicing of multiple similar introns of different bacterial genes. J Biol Chem. 2005; 280:35085–8. 16150738 10.1074/jbc.C500328200

[53] Stoddard BL Homing endonucleases from mobile group I introns: discovery to genome engineering. Mobile DNA. 2014; 5:7. 24589358 10.1186/1759-8753-5-7 PMC3943268

[54] Mullineux ST, Costa M, Bassi GS et al. A group II intron encodes a functional LAGLIDADG homing endonuclease and self-splices under moderate temperature and ionic conditions. RNA. 2010; 16:1818–31. 20656798 10.1261/rna.2184010 PMC2924541

[55] Abramson J, Adler J, Dunger J et al. Accurate structure prediction of biomolecular interactions with AlphaFold 3. Nature. 2024; 630:493–500. 38718835 10.1038/s41586-024-07487-w PMC11168924

[56] Dassa B, London N, Stoddard BL et al. Fractured genes: a novel genomic arrangement involving new split inteins and a new homing endonuclease family. Nucleic Acids Res. 2009; 37:2560–73. 19264795 10.1093/nar/gkp095 PMC2677866

[57] Sitbon E, Pietrokovski S New types of conserved sequence domains in DNA-binding regions of homing endonucleases. Trends Biochem Sci. 2003; 28:473–7. 13678957 10.1016/S0968-0004(03)00170-1

[58] Yutin N, Benler S, Shmakov SA et al. Analysis of metagenome-assembled viral genomes from the human gut reveals diverse putative CrAss-like phages with unique genomic features. Nat Commun. 2021; 12:1044. 33594055 10.1038/s41467-021-21350-w PMC7886860

[59] Yutin N, Tolstoy I, Mutz P et al. Jumping DNA polymerases in bacteriophages. bioRxiv27 April 2024, preprint: not peer reviewed 10.1101/2024.04.26.591309.

