# Supplementary figures and images for: Presence of group II introns in phage genomes
Source: Nucleic Acids Res. 2025 Aug 13;53(15):gkaf761. doi: 10.1093/nar/gkaf761 (PMC12350094; doi:10.1093/nar/gkaf761)

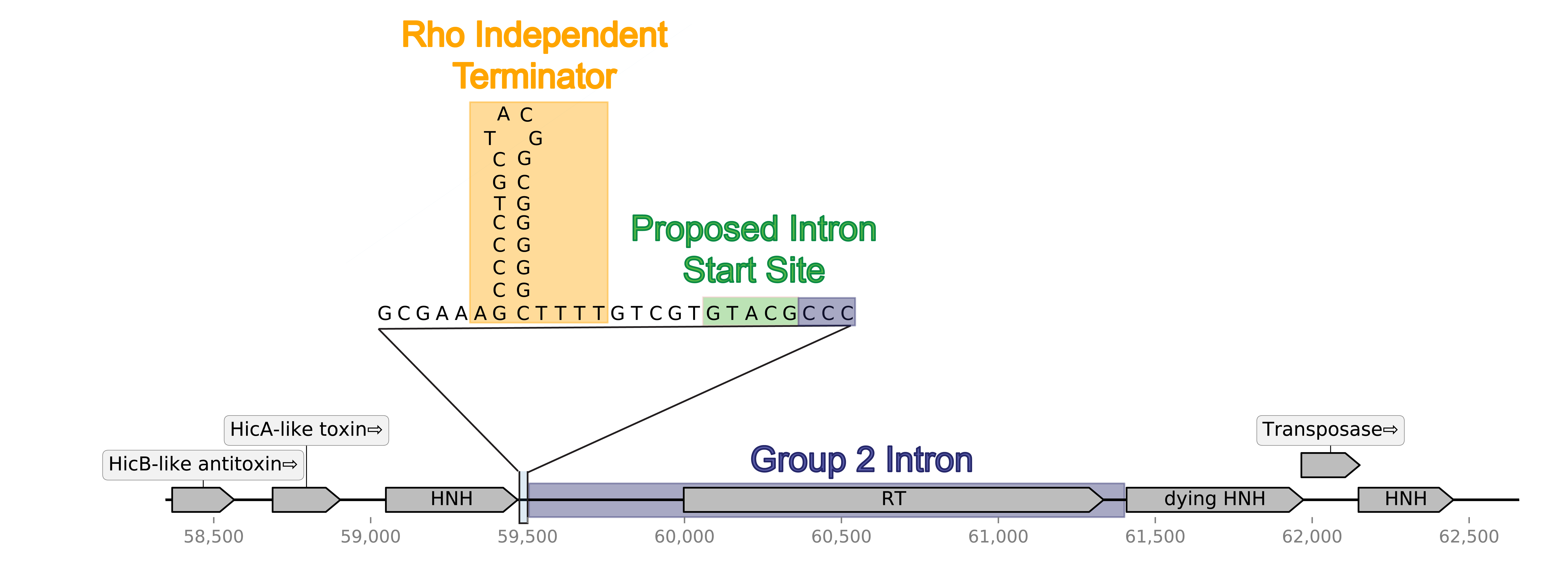

Supplement: gkaf761_Supplemental_Files [file gkaf761_supplemental_files.zip › supp_fig1.png]
